# Synthesis and crystal structure of (*E*)-2-benzyl-1,3-di­phenyl­iso­thio­uronium iodide

**DOI:** 10.1107/S2056989021013086

**Published:** 2022-01-01

**Authors:** Sungmin Kang, Taek Hyeon Kim, Chee-Hun Kwak

**Affiliations:** a School of Chemical Engineering, College of Engineering, Chonnam National, University, Gwangju, 61186, South Korea; b Department of Chemistry Education, Sunchon National University, 255 Jungang-ro, Sunchon, 57922, South Korea

**Keywords:** crystal structure, thio­ether, N—H⋯I hydrogen bonding

## Abstract

In the title mol­ecular salt, C_20_H_19_N_2_S^+^·I^−^, prepared by the reaction of 1,3-di­phenyl­thio­urea and benzyl iodide, the C—S—C thio­ether bond angle is 101.66 (9)° and electrons are delocalized over the N^+^= C—N skeleton. The dihedral angle between the aromatic rings attached to the N atoms is 40.60 (9)°. In the crystal, N—H⋯I hydrogen bonds link the components into [100] chains.

## Abstract 2

The title compound, a salt form of (*E*)-2-benzyl-1,3-di­phenyl­iso­thio­uronium iodide, was prepared by the reaction of 1,3-di­phenyl­thio­urea and benzyl iodide. In the crystal, N—H⋯I hydrogen bonds link the components into [100] chains.

## Chemical context

Iso­thio­uronium salts containing an *R*–S–C–(NH*R*)_2_^+^ moiety have been investigated as their hydrogen–bonding motifs for mol­ecular recognition of anions (Yeo & Hong, 1998 ▸; Kubo *et al.*, 2000 ▸; Kato *et al.*, 2004 ▸; Nguyen *et al.*, 2009 ▸; Nguyen & Kim, 2010 ▸) and as organocatalysts (Nguyen & Kim, 2011 ▸, 2012 ▸; Lee *et al.*, 2018 ▸; Kang *et al.*, 2019 ▸). The iso­thio­uronium group could enhance the acidity of their NH groups compared with thio­urea and therefore be used as prospective alternative for thio­urea. In addition, the chemical modification of the iso­thio­uronium skeleton is readily performed using alkyl­ation reactions of thio­urea. As part of our work in this area, the synthesis and single-crystal structure of the title mol­ecular salt, C_20_H_19_N_2_S^+^·I^−^ are reported herein. 
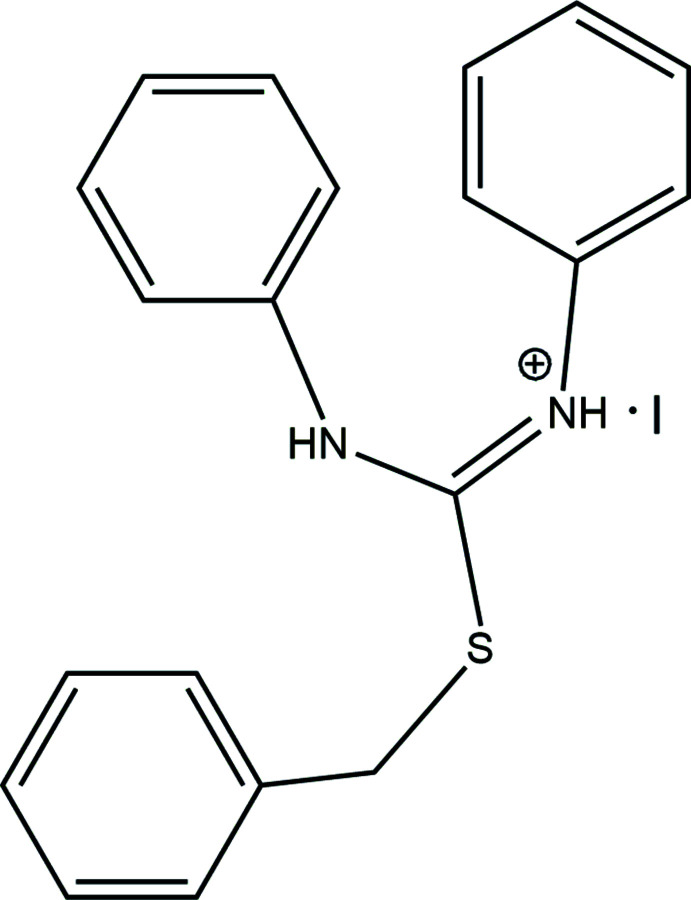


## Structural commentary

**Figure 1:**
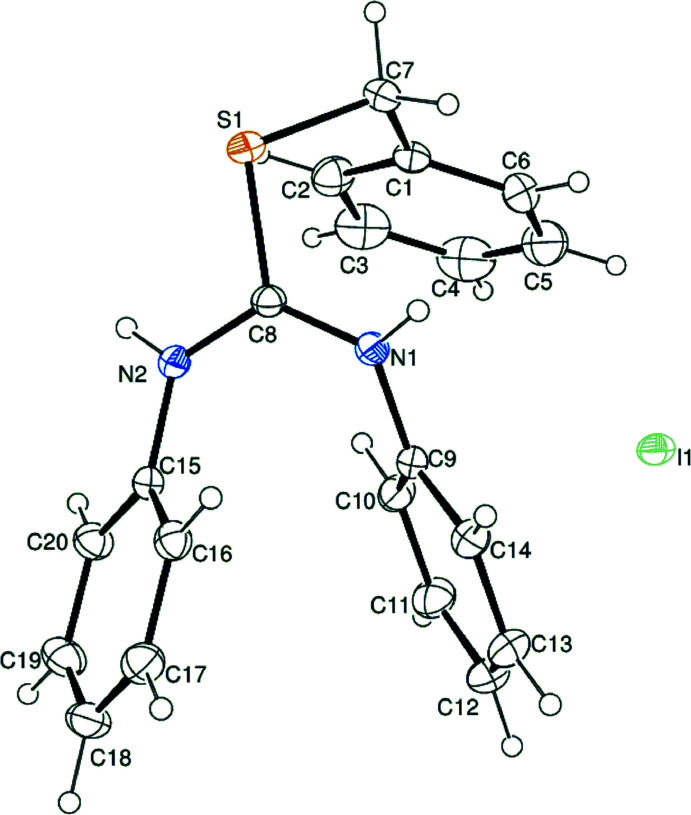
The mol­ecular structure of the title compound with displacement ellipsoids drawn at at the 30% probability level.

The title compound, C_20_H_19_N_2_S^+^·I^−^ (Fig. 1 ▸), is a mol­ecular salt that arose from the reaction of 1,3-di­phenyl­thio­urea and benzyl iodide. There are three benzene rings, C1–C6 (I), C9–C14 (II) and C15–C20 (III) in the cation and the dihedral angles I/II, II/III and I/III are 50.36 (8), 40.60 (9) and 85.45 (9)°, respectively. In the cation, the *N*-[(phenyl­amino)­methyl­ene]benzenaminium and toluyl units are linked to the sulfur atom as a thio­ether. The C7—S1 and C8—S1 bond lengths are 1.823 (2) and 1.751 (2) Å, respectively, and the C—S—C bond angle is 101.66 (9)°. The conformation of C1 and C8 about the C7—S1 bond is *gauche* [C1—C7—S1—C8 = 49.53 (16)°]. The C—S—C bond angle in the title compound is somewhat smaller than that for di-*p*-tolyl sulfide (109°; Blackmore & Abrahams, 1955 ▸) or the angle (107.8°) in oligomeric [ArCOArSArCOAr] (Ar = 1,4-phenyl­ene; Colquhoun *et al.*, 1999 ▸) in which the aromatic rings are nearly coplanar. Rather, it is closer to that seen in diethyl sulfide [99.05 (4)°; Iijima *et al.*, 1977 ▸]. This result can be explained by the large dihedral angle between the benzene rings in the title compound. In the *N*-[(phenyl­amino)­methyl­ene]benz­en­aminium moiety of the title cation, the π-electrons of the iminium double bond are delocalized over the N1—C6—N2 skeleton [the C8—N1 and C8—N2 bond distances are 1.319 (2) and 1.332 (2) Å, respectively, and N1—C8—N2 = 124.53 (16)°].

## Supra­molecular features

**Figure 2:**
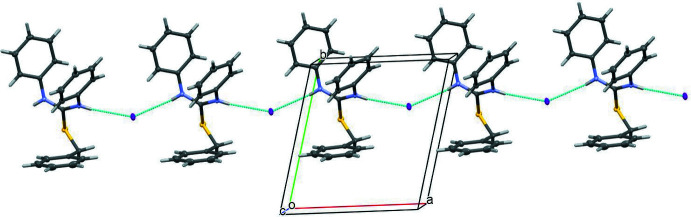
A view of a fragment of the [100] chain arising from N—H⋯I hydrogen bonds.

In the crystal, the cations and anions are linked by almost linear N—H⋯I hydrogen bonds (Fig. 2 ▸, Table 1 ▸), generating [100] chains of alternating cations and anions, with adjacent species in the chain related by simple translation. No significant aromatic π–π stacking inter­actions occur, the shortest centroid–centroid separation being greater than 4.7 Å.

## Database survey

A search of the Cambridge Structural Database (CSD, *via* CCDC Access Structures, November 2021; Groom *et al.*, 2016 ▸) resulted in 30 structures using iso­thio­uronium as the keyword: 26 of them have a thio­ether skeleton. No results were found for 2-benzyl-1,3-di­phenyl­iso­thio­uronium or *N*-[(phenyl­amino)­methyl­ene]benzenaminium but the compound most similar to the title compound is *S*-benzyl­iso­thio­uronium chloride (Barker & Powell, 1998 ▸). The bond angles of the thio­ether group in the *S*-benzyl­iso­thio­uronium salts similar to the title compound are the range 102.6 to 104.8°, depending on the counter-anions (Hemalatha & Veeravazhuthi, 2008 ▸; Ishii *et al.*, 2000 ▸; Pope & Boeyens, 1975 ▸).

## Synthesis and crystallization

1,3-Di­phenyl­thio­urea (4.4 mmol) was added to a solution of benzyl iodide (13.2 mmol) in dry di­chloro­methane at room temperature. The reaction mixture was then stirred for 24 h and concentrated *in vacuo*. The residue was purified *via* flash chromatography (hexa­ne:ethyl acetate = 8:2), to give a the title compound as a yellow solid (1.14 g, yield 58%). A solution of iso­thio­uronium iodide in methanol was slowly evap­orated at room temperature to give crystals of the title compound: m.p. 442–443 K; ^1^H NMR (300 MHz, DMSO): δ 7.21–7.39 (*m*, 15 H), δ 4.45 (*s*, 2 H); HR TOF–MS for C_20_H_18_N_2_S: calculated 318.1186 (*M* ^+^), found 318.1185 (*M* ^+^).

## Refinement

Crystal data, data collection and structure refinement details are summarized in Table 2 ▸. H atoms were positioned geometrically (C—H = 0.94–0.98 Å, N—H = 0.80 Å) and refined using a riding model with*U*_iso_(H) = 1.2*U*_eq_(carrier).

## Supplementary Material

Crystal structure: contains datablock(s) I. DOI: 10.1107/S2056989021013086/hb7997sup1.cif

Structure factors: contains datablock(s) I. DOI: 10.1107/S2056989021013086/hb7997Isup2.hkl

Supporting information file. DOI: 10.1107/S2056989021013086/hb7997Isup3.cml

CCDC reference: 2127354

Additional supporting information:  crystallographic information; 3D view; checkCIF report

## Figures and Tables

**Table 1:** Hydrogen-bond geometry (Å, °)

*D*—H⋯*A*	*D*—H	H⋯*A*	*D*⋯*A*	*D*—H⋯*A*
N1—H1*N*⋯I1^i^	0.80 (3)	2.69 (3)	3.4781 (17)	171 (2)
N2—H2*N*⋯I1^ii^	0.80 (3)	2.73 (3)	3.5242 (17)	169 (2)

Symmetry codes: (i) -x+1, -y+1, -z+1; (ii) -x, -y+1, -z+1.

**Table 2:** Experimental details

Crystal data	
Chemical formula	C_20_H_19_N_2_S^+^·I^−^
*M*_r_	446.33
Crystal system, space group	Triclinic, *P*\overline{1}
Temperature (K)	223
*a*, *b*, *c* (Å)	8.6382 (3), 9.8182 (3), 12.1922 (4)
α, β, γ (°)	77.2839 (12), 85.1708 (11), 74.7224 (10)
*V* (Å^3^)	972.66 (6)
*Z*	2
Radiation type	Mo *K*α
μ (mm^−1^)	1.76
Crystal size (mm)	0.27 × 0.21 × 0.15

Data collection	
Diffractometer	PHOTON 100 CMOS
Absorption correction	Multi-scan (*SADABS*; Bruker, 2016 ▸)
*T*_min_, *T*_max_	0.649, 0.746
No. of measured, independent and observed [*I* > 2σ(*I*)] reflections	31969, 4853, 4594
*R*_int_	0.023
(sin θ/λ)_max_ (Å^−1^)	0.668

Refinement	
*R*[*F* ^2^ > 2σ(*F* ^2^)], *wR*(*F* ^2^), *S*	0.025, 0.063, 1.09
No. of reflections	4853
No. of parameters	225
H-atom treatment	H atoms treated by a mixture of independent and constrained refinement
Δρ_max_, Δρ_min_ (e Å^−3^)	1.43, −1.04

Computer programs: *APEX2* and *SAINT* (Bruker, 2016 ▸), *SHELXTL* (Sheldrick, 2008 ▸), *SHELXL2014/7* (Sheldrick, 2015 ▸), *ORTEP-3 for Windows* (Farrugia, 2012 ▸) and *Mercury* (Macrae *et al.*, 2020 ▸).

## supplementary crystallographic information

### Crystal data

**Table:**

C_20_H_19_N_2_S^+^·I^−^	*Z* = 2
*M**_r_* = 446.33	*F*(000) = 444
Triclinic, *P*1	*D*_x_ = 1.524 Mg m^−^^3^
*a* = 8.6382 (3) Å	Mo *K*α radiation, λ = 0.71073 Å
*b* = 9.8182 (3) Å	Cell parameters from 9837 reflections
*c* = 12.1922 (4) Å	θ = 2.5–28.3°
α = 77.2839 (12)°	µ = 1.76 mm^−^^1^
β = 85.1708 (11)°	*T* = 223 K
γ = 74.7224 (10)°	Block, colourless
*V* = 972.66 (6) Å^3^	0.27 × 0.21 × 0.15 mm

### Data collection

**Table:**

PHOTON 100 CMOS diffractometer	4594 reflections with *I* > 2σ(*I*)
φ and ω scans	*R*_int_ = 0.023
Absorption correction: multi-scan (SADABS; Bruker, 2016)	θ_max_ = 28.3°, θ_min_ = 2.2°
*T*_min_ = 0.649, *T*_max_ = 0.746	*h* = −11→11
31969 measured reflections	*k* = −13→13
4853 independent reflections	*l* = −16→16

### Refinement

**Table:**

Refinement on *F*^2^	Primary atom site location: structure-invariant direct methods
Least-squares matrix: full	Hydrogen site location: mixed
*R*[*F*^2^ > 2σ(*F*^2^)] = 0.025	H atoms treated by a mixture of independent and constrained refinement
*wR*(*F*^2^) = 0.063	*w* = 1/[σ^2^(*F*_o_^2^) + (0.0253*P*)^2^ + 0.8267*P*] where *P* = (*F*_o_^2^ + 2*F*_c_^2^)/3
*S* = 1.09	(Δ/σ)_max_ = 0.001
4853 reflections	Δρ_max_ = 1.43 e Å^−^^3^
225 parameters	Δρ_min_ = −1.03 e Å^−^^3^
0 restraints	

### Special details

**Table:**

Geometry. All esds (except the esd in the dihedral angle between two l.s. planes) are estimated using the full covariance matrix. The cell esds are taken into account individually in the estimation of esds in distances, angles and torsion angles; correlations between esds in cell parameters are only used when they are defined by crystal symmetry. An approximate (isotropic) treatment of cell esds is used for estimating esds involving l.s. planes.

### Fractional atomic coordinates and isotropic or equivalent isotropic displacement parameters (Å^2^)

**Table:**

	*x*	*y*	*z*	*U*_iso_*/*U*_eq_	
I1	0.26986 (2)	0.34036 (2)	0.87755 (2)	0.04395 (6)	
C1	0.3315 (2)	0.4161 (2)	0.28202 (17)	0.0316 (4)	
C2	0.1753 (3)	0.4055 (3)	0.3116 (2)	0.0421 (5)	
H2	0.1020	0.4181	0.2552	0.051*	
C3	0.1274 (4)	0.3766 (3)	0.4237 (2)	0.0581 (7)	
H3	0.0216	0.3697	0.4430	0.070*	
C4	0.2340 (5)	0.3578 (3)	0.5072 (2)	0.0648 (8)	
H4	0.2012	0.3380	0.5833	0.078*	
C5	0.3880 (4)	0.3682 (3)	0.4786 (2)	0.0594 (7)	
H5	0.4609	0.3550	0.5355	0.071*	
C6	0.4373 (3)	0.3982 (2)	0.3666 (2)	0.0434 (5)	
H6	0.5427	0.4064	0.3479	0.052*	
C7	0.3883 (2)	0.4451 (2)	0.16100 (17)	0.0330 (4)	
H7A	0.4058	0.3566	0.1320	0.040*	
H7B	0.4917	0.4696	0.1574	0.040*	
S1	0.24895 (6)	0.59022 (5)	0.07051 (4)	0.03231 (10)	
C8	0.2092 (2)	0.72734 (19)	0.14740 (14)	0.0252 (3)	
N1	0.32455 (19)	0.75297 (18)	0.19789 (14)	0.0278 (3)	
H1N	0.415 (3)	0.724 (3)	0.177 (2)	0.036 (6)*	
C9	0.3042 (2)	0.82793 (19)	0.28816 (15)	0.0267 (3)	
C10	0.2028 (2)	0.7956 (2)	0.37864 (17)	0.0337 (4)	
H10	0.1474	0.7243	0.3814	0.040*	
C11	0.1841 (3)	0.8703 (3)	0.46533 (18)	0.0426 (5)	
H11	0.1147	0.8502	0.5270	0.051*	
C12	0.2668 (3)	0.9740 (3)	0.46136 (19)	0.0444 (5)	
H12	0.2526	1.0250	0.5198	0.053*	
C13	0.3700 (3)	1.0030 (2)	0.3723 (2)	0.0421 (5)	
H13	0.4272	1.0727	0.3708	0.051*	
C14	0.3903 (2)	0.9300 (2)	0.28442 (18)	0.0343 (4)	
H14	0.4612	0.9493	0.2236	0.041*	
N2	0.05644 (19)	0.80281 (17)	0.14673 (14)	0.0276 (3)	
H2N	−0.010 (3)	0.765 (3)	0.134 (2)	0.034 (6)*	
C15	−0.0057 (2)	0.94592 (19)	0.16504 (15)	0.0259 (3)	
C16	0.0742 (2)	1.0527 (2)	0.12339 (16)	0.0310 (4)	
H16	0.1723	1.0316	0.0832	0.037*	
C17	0.0078 (3)	1.1909 (2)	0.14165 (18)	0.0380 (4)	
H17	0.0622	1.2636	0.1148	0.046*	
C18	−0.1379 (3)	1.2225 (2)	0.19912 (19)	0.0422 (5)	
H18	−0.1816	1.3161	0.2122	0.051*	
C19	−0.2187 (3)	1.1169 (2)	0.2371 (2)	0.0427 (5)	
H19	−0.3192	1.1396	0.2743	0.051*	
C20	−0.1536 (2)	0.9773 (2)	0.22119 (18)	0.0349 (4)	
H20	−0.2086	0.9051	0.2479	0.042*	

### Atomic displacement parameters (Å^2^)

**Table:**

	*U*^11^	*U*^22^	*U*^33^	*U*^12^	*U*^13^	*U*^23^
I1	0.02782 (7)	0.04822 (9)	0.06798 (11)	−0.01720 (6)	0.01189 (6)	−0.03314 (7)
C1	0.0347 (10)	0.0226 (8)	0.0376 (10)	−0.0046 (7)	−0.0028 (8)	−0.0091 (7)
C2	0.0428 (12)	0.0421 (11)	0.0462 (12)	−0.0177 (9)	0.0024 (9)	−0.0122 (9)
C3	0.0661 (17)	0.0529 (15)	0.0582 (16)	−0.0274 (13)	0.0190 (13)	−0.0102 (12)
C4	0.099 (2)	0.0473 (15)	0.0396 (13)	−0.0139 (15)	0.0082 (14)	−0.0006 (11)
C5	0.079 (2)	0.0483 (14)	0.0424 (13)	0.0001 (13)	−0.0211 (13)	−0.0048 (11)
C6	0.0419 (12)	0.0377 (11)	0.0474 (12)	0.0001 (9)	−0.0127 (10)	−0.0102 (9)
C7	0.0294 (9)	0.0298 (9)	0.0393 (10)	−0.0018 (7)	−0.0002 (8)	−0.0136 (8)
S1	0.0365 (2)	0.0321 (2)	0.0301 (2)	−0.00373 (18)	−0.00340 (18)	−0.01509 (18)
C8	0.0254 (8)	0.0257 (8)	0.0253 (8)	−0.0059 (6)	0.0010 (6)	−0.0083 (6)
N1	0.0199 (7)	0.0326 (8)	0.0330 (8)	−0.0045 (6)	0.0014 (6)	−0.0146 (6)
C9	0.0231 (8)	0.0283 (8)	0.0294 (8)	−0.0030 (6)	−0.0050 (6)	−0.0104 (7)
C10	0.0331 (9)	0.0385 (10)	0.0329 (9)	−0.0115 (8)	−0.0011 (7)	−0.0117 (8)
C11	0.0459 (12)	0.0521 (13)	0.0331 (10)	−0.0119 (10)	0.0023 (9)	−0.0172 (9)
C12	0.0509 (13)	0.0452 (12)	0.0415 (11)	−0.0055 (10)	−0.0079 (10)	−0.0237 (10)
C13	0.0450 (12)	0.0372 (11)	0.0514 (13)	−0.0139 (9)	−0.0106 (10)	−0.0167 (9)
C14	0.0322 (9)	0.0348 (10)	0.0393 (10)	−0.0110 (8)	−0.0029 (8)	−0.0106 (8)
N2	0.0229 (7)	0.0292 (8)	0.0338 (8)	−0.0063 (6)	−0.0039 (6)	−0.0122 (6)
C15	0.0253 (8)	0.0262 (8)	0.0257 (8)	−0.0028 (6)	−0.0054 (6)	−0.0070 (6)
C16	0.0321 (9)	0.0324 (9)	0.0274 (8)	−0.0074 (7)	−0.0021 (7)	−0.0043 (7)
C17	0.0489 (12)	0.0300 (9)	0.0353 (10)	−0.0118 (9)	−0.0066 (9)	−0.0025 (8)
C18	0.0523 (13)	0.0287 (10)	0.0416 (11)	0.0008 (9)	−0.0057 (9)	−0.0108 (8)
C19	0.0368 (11)	0.0405 (11)	0.0460 (12)	0.0010 (9)	0.0052 (9)	−0.0143 (9)
C20	0.0300 (9)	0.0342 (10)	0.0406 (10)	−0.0071 (8)	0.0024 (8)	−0.0105 (8)

### Geometric parameters (Å, º)

**Table:**

C1—C6	1.386 (3)	C10—C11	1.390 (3)
C1—C2	1.392 (3)	C10—H10	0.9400
C1—C7	1.505 (3)	C11—C12	1.381 (3)
C2—C3	1.383 (4)	C11—H11	0.9400
C2—H2	0.9400	C12—C13	1.374 (4)
C3—C4	1.381 (5)	C12—H12	0.9400
C3—H3	0.9400	C13—C14	1.391 (3)
C4—C5	1.371 (5)	C13—H13	0.9400
C4—H4	0.9400	C14—H14	0.9400
C5—C6	1.388 (4)	N2—C15	1.426 (2)
C5—H5	0.9400	N2—H2N	0.81 (3)
C6—H6	0.9400	C15—C16	1.386 (3)
C7—S1	1.823 (2)	C15—C20	1.391 (3)
C7—H7A	0.9800	C16—C17	1.386 (3)
C7—H7B	0.9800	C16—H16	0.9400
S1—C8	1.7513 (18)	C17—C18	1.383 (3)
C8—N1	1.319 (2)	C17—H17	0.9400
C8—N2	1.332 (2)	C18—C19	1.376 (4)
N1—C9	1.428 (2)	C18—H18	0.9400
N1—H1N	0.80 (3)	C19—C20	1.388 (3)
C9—C10	1.384 (3)	C19—H19	0.9400
C9—C14	1.388 (3)	C20—H20	0.9400
			
C6—C1—C2	118.9 (2)	C9—C10—H10	120.5
C6—C1—C7	119.46 (19)	C11—C10—H10	120.5
C2—C1—C7	121.67 (19)	C12—C11—C10	120.3 (2)
C3—C2—C1	120.3 (2)	C12—C11—H11	119.9
C3—C2—H2	119.8	C10—C11—H11	119.9
C1—C2—H2	119.8	C13—C12—C11	120.3 (2)
C4—C3—C2	120.4 (3)	C13—C12—H12	119.9
C4—C3—H3	119.8	C11—C12—H12	119.9
C2—C3—H3	119.8	C12—C13—C14	120.5 (2)
C5—C4—C3	119.6 (3)	C12—C13—H13	119.8
C5—C4—H4	120.2	C14—C13—H13	119.8
C3—C4—H4	120.2	C9—C14—C13	118.8 (2)
C4—C5—C6	120.6 (3)	C9—C14—H14	120.6
C4—C5—H5	119.7	C13—C14—H14	120.6
C6—C5—H5	119.7	C8—N2—C15	127.80 (16)
C1—C6—C5	120.2 (2)	C8—N2—H2N	117.4 (18)
C1—C6—H6	119.9	C15—N2—H2N	114.8 (18)
C5—C6—H6	119.9	C16—C15—C20	120.73 (17)
C1—C7—S1	113.83 (13)	C16—C15—N2	121.30 (17)
C1—C7—H7A	108.8	C20—C15—N2	117.89 (17)
S1—C7—H7A	108.8	C17—C16—C15	119.28 (19)
C1—C7—H7B	108.8	C17—C16—H16	120.4
S1—C7—H7B	108.8	C15—C16—H16	120.4
H7A—C7—H7B	107.7	C18—C17—C16	120.3 (2)
C8—S1—C7	101.66 (9)	C18—C17—H17	119.8
N1—C8—N2	124.53 (16)	C16—C17—H17	119.8
N1—C8—S1	121.30 (14)	C19—C18—C17	120.0 (2)
N2—C8—S1	114.14 (13)	C19—C18—H18	120.0
C8—N1—C9	126.22 (16)	C17—C18—H18	120.0
C8—N1—H1N	118.0 (19)	C18—C19—C20	120.7 (2)
C9—N1—H1N	115.7 (19)	C18—C19—H19	119.7
C10—C9—C14	121.18 (18)	C20—C19—H19	119.7
C10—C9—N1	119.87 (17)	C19—C20—C15	118.9 (2)
C14—C9—N1	118.93 (17)	C19—C20—H20	120.5
C9—C10—C11	119.0 (2)	C15—C20—H20	120.5
			
C6—C1—C2—C3	−0.5 (3)	C9—C10—C11—C12	−0.6 (3)
C7—C1—C2—C3	179.0 (2)	C10—C11—C12—C13	−0.8 (4)
C1—C2—C3—C4	0.0 (4)	C11—C12—C13—C14	1.0 (4)
C2—C3—C4—C5	0.1 (4)	C10—C9—C14—C13	−1.7 (3)
C3—C4—C5—C6	0.3 (4)	N1—C9—C14—C13	179.86 (18)
C2—C1—C6—C5	0.9 (3)	C12—C13—C14—C9	0.3 (3)
C7—C1—C6—C5	−178.6 (2)	N1—C8—N2—C15	21.8 (3)
C4—C5—C6—C1	−0.8 (4)	S1—C8—N2—C15	−156.34 (15)
C6—C1—C7—S1	−134.43 (17)	C8—N2—C15—C16	38.5 (3)
C2—C1—C7—S1	46.1 (2)	C8—N2—C15—C20	−144.78 (19)
C1—C7—S1—C8	49.53 (16)	C20—C15—C16—C17	2.2 (3)
C7—S1—C8—N1	41.74 (18)	N2—C15—C16—C17	178.92 (17)
C7—S1—C8—N2	−140.01 (15)	C15—C16—C17—C18	−1.1 (3)
N2—C8—N1—C9	22.1 (3)	C16—C17—C18—C19	−0.9 (3)
S1—C8—N1—C9	−159.85 (15)	C17—C18—C19—C20	1.8 (4)
C8—N1—C9—C10	46.0 (3)	C18—C19—C20—C15	−0.7 (3)
C8—N1—C9—C14	−135.6 (2)	C16—C15—C20—C19	−1.4 (3)
C14—C9—C10—C11	1.9 (3)	N2—C15—C20—C19	−178.16 (18)
N1—C9—C10—C11	−179.70 (19)		

### Hydrogen-bond geometry (Å, º)

**Table:**

*D*—H···*A*	*D*—H	H···*A*	*D*···*A*	*D*—H···*A*
N1—H1*N*···I1^i^	0.80 (3)	2.69 (3)	3.4781 (17)	171 (2)
N2—H2*N*···I1^ii^	0.80 (3)	2.73 (3)	3.5242 (17)	169 (2)

Symmetry codes: (i) −*x*+1, −*y*+1, −*z*+1; (ii) −*x*, −*y*+1, −*z*+1.
